# COVID-19, cardiovascular diseases and cardiac troponins

**DOI:** 10.2217/fca-2021-0054

**Published:** 2021-09-03

**Authors:** Chan W Kim, Wilbert S Aronow

**Affiliations:** ^1^Cardiology Department, & The Department of Medicine, Westchester Medical Center & New York Medical College, Valhalla, NY 10595, USA

**Keywords:** cardiac troponin, cardiovascular diseases, COVID-19

## Abstract

There has been strong evidence of myocardial injury in coronavirus disease 2019 (COVID-19) patients with significantly elevated serum cardiac troponin (cTn). While the exact mechanism of injury is unclear, possible suggested pathological mechanisms of injury are discussed. These include increased susceptibility of the myocardium and endothelium to viral invasion, underlying hyperinflammatory state and subsequent cytokine storm, a hypercoagulable and prothrombotic state, and indirect myocardial injury due to hypoxemia. As a result of these pathological mechanisms in COVID-19 patients, cTn may be elevated largely due to myocarditis, microangiopathy or myocardial infarction. The utility of cTn as a biomarker for measuring myocardial injury in these patients and assessing its ability as a prognostic factor for clinical outcome is also discussed.

Cardiovascular diseases (CVD) and circulatory diseases are currently recognized as the leading causes of mortality in the world accounting for 32% of global deaths [1,2]. Despite the decrease in death rates over the years, the number of CVD deaths have been rising, largely due to both the growth and the aging of the global population, and while the global age-standardized rate of CVD deaths decreased from 376 per 100,000 in 1990 to 293 per 100,000 in 2013, the number of CVD deaths increased from 12.3 to 17.3 million [1,3,4]. Furthermore, the increase in the prevalence of CVD is a global issue and must be taken into consideration with the current coronavirus disease 2019 (COVID-19) epidemic. In the current COVID-19 epidemic, caused by severe acute respiratory syndrome coronavirus 2 (SARS-CoV-2) infection, as of 23 February 2021, according to weekly epidemiological update by the WHO, globally, there have been over 2.4 million new cases and 66,000 new deaths reported, bringing the global cumulative numbers to over 103 million reported cases and 1.7 million deaths globally [5–7]. Additionally, the United States has over 28 million reported cases and over 500,000 deaths. While the overall mortality rate remains relatively low from 1.4 to 2.3%, there has been significant evidence that the risk of mortality is significantly higher in patients with comorbidities such as Type 2 diabetes mellitus, hypertension (HTN), CVD, cancer, chronic kidney disease, congestive heart failure, obesity and smoking [8–11]. These comorbidities have all shown to be associated with increased severity of COVID-19 infection, with severity defined as hospitalization, intensive care, intubation, mechanical ventilation or death. Furthermore, while COVID-19 is an infection primarily affecting the respiratory system, studies have shown that COVID-19 infection is associated with worsening of underlying CVD and possible induction of cardiac pathologies that were not present prior to the infection [12,13]. Therefore, the purpose of this review article is to discuss CVD as a risk factor for severe COVID-19 illness, to elucidate the pathogenesis of the interaction between infection by SARS-CoV-2, myocardial injury, and elevated cardiac troponin (cTn), and to explore the potential of cTn as a marker of myocardial damage in predicting the health outcomes of COVID-19 patients. This analysis is fundamental as it can perhaps provide insight into the global vulnerability of patients to COVID-19; as the number of patients with COVID-19 grows so too will the number of potential patients with underlying comorbidities that can result in critical illness that requires an intensive level of care.

## Cardiovascular health & COVID-19

According to reports from China during the earlier days of the COVID-19 pandemic, pre-existing CVD has been indicated to be associated with a worse prognosis following infection with SARS-CoV-2. For example, a retrospective study of 187 patients with confirmed COVID-19 in Wuhan showed that 27.8% of patients had myocardial injury, and eventually suffered cardiac dysfunction and arrhythmias [14]. A separate study of COVID-19 patients in Wuhan additionally reported that those who developed acute respiratory distress syndromes (ARDS) commonly shared comorbidities such as HTN and diabetes [15]. Overall, early studies of COVID-19 disease severity and case fatality rates have emphasized the development of critical illness for pre-existing comorbid conditions such as CVD, HTN, coronary artery disease and heart failure, reporting up to a fivefold increase in death rate attributable to pre-existing cardiovascular complications. Naturally, such reports underline the importance of recognizing the worse prognoses of these patient populations [15,16]. Subsequent studies of COVID-19 patients have similarly reported the association between CVD and higher rates of critical intensive care unit (ICU) care and mortality, and a case-series study of 6700 patients in the New York City area reported that HTN was the most frequent comorbidity at 56.6% [17–21]. The link between pre-existing HTN and the increased risk of developing severe disease following SARS-CoV-2 infection has been a point of interest in studying the implications of COVID-19 on cardiovascular health [22,23]. It is also important to note that the increased prevalence of HTN in patients who have worse outcomes must be taken in consideration along with the fact that these patients are often the elderly [24]. Furthermore, these patients already experience a natural state of inflammation due to their health, and it is suggested that the SARS-CoV-2 virus interacts with this inflammatory state to induce the decrease in cardiac reserve and cause worse outcomes [25,26]. Studies have additionally shown how the infection with COVID-19 induces damage to the myocardium resulting in inflammation and damage, and myocardial injuries are associated with increased incidences of ARDS, coagulopathy, and need of ventilation, both noninvasive and invasive [27,28].

Regarding the health of the patient’s myocardium, increased levels of cardiomyocyte structural protein, cTn, in plasma has been shown to be frequently correlated with higher rates of complications and mortality [14]. Relatedly, higher levels of cTn were correlated with malignant arrhythmias, and mortality rates of patients receiving or not receiving ACE inhibitors or angiotensin receptor blockers suggested that while preexisting cardiovascular damage may yield more severe COVID-19 illness, there is likely no increased susceptibility to contracting the virus itself [28]. Other studies have also described a strong correlation between cTn and other inflammatory factors such as C-reactive protein (CRP) with increased rates of mechanical ventilation and ventricular and atrial arrhythmias, likely due to myocardial inflammation involving the electrical circuits of myocytes [29]. Furthermore, additional reports from Wuhan showed that the risk of COVID-19 severity was significantly increased for patients with age ≥65 years, pre-existing CVD, decreased T-cells counts and elevated cardiac troponin I (cTnI) [30]. In a multicenter retrospective study of 607 COVID-19 patients in Istanbul, Turkey, laboratory results expectedly showed significant increases in the levels of inflammatory factors, such as CRP, procalcitonin, D-dimer, aspartate transaminase and high sensitivity cardiac troponin I (hs-cTnI), in patients who required ICU care, those who developed ARDS, and those who died [31]. Ultimately, studies throughout the COVID-19 pandemic emphasize the association between cardiovascular health and disease prognosis, and thus it is critical to recognize the impact of this common comorbidity on the vulnerable population.

## Pathogenesis of elevated troponin in COVID-19

Myocardial injury is indicated by an increase in cTn levels and can be the result of multiple pathogenesis including but not limited to hypoxia, systemic inflammation, thrombosis, embolism or sepsis. Elevated levels of cTn is common in severe cases of COVID-19 disease and in non-COVID-19 critically ill patients [32,33]. While the exact pathophysiology of elevated cTn in COVID-19 has not yet been fully elucidated, there have been many studies providing insight into different mechanisms of injury to the myocardium in these patients. Most simply, the pathophysiology of elevated cTn can be approached as whether the damage to the myocardium is a primary disease due to the virus with direct entry into the myocardial cells or endothelial cells or a secondary disease through damage to other organ systems resulting in myocardial damage.

The key underlying process for myocardial injury and one of the pathophysiological hallmarks of COVID-19 is the hyperactivation of the immune system with a prominent IL-6 response, resulting in severe inflammation and a cytokine storm, consequently causing systemic damage [34–36]. An important topic of study has been angiotensin-converting enzyme 2 (ACE2) and its role in COVID-19 infection and management. ACE2 has been identified as the point of host cell entry by SARS-CoV-2, and infection occurs via the coronavirus spike proteins, which are activated into a receptor-binding domain and a membrane-fusion domain following cleavage by host cell proteases [37–39]. Because ACE2 is a target for SARS-CoV-2, a natural possibility is that cells with the greatest expression of ACE2 are the most vulnerable to infection. Thus, in the human body, the areas that are among the most at risk are the lungs, heart, vasculature endothelium, gastrointestinal tract epithelium and kidneys. ACE2 is also involved in the catalysis of Angiotensin (Ang) II to Ang 1–7. Ang 1–7 are involved in protecting the cardiovascular system by opposing the vasoconstrictive, pro-inflammatory, proproliferative and profibrotic actions of Ang II [40]. Therefore, the myocardium and endothelium are more susceptible to SARS-CoV-2 due to ACE2 expression, and by the virus binding to ACE2, physiologic level of ACE2 is decreased, reducing the production of Ang 1–7, ultimately decreasing the protective effect on the cardiovascular system.

In a combination of the underlying chemokine storm affecting the entire body, SARS-CoV-2 preference for the myocardium and endothelium, and the inhibitory effects on ACE2 and Ang 1–7, myocardial injury can occur via multiple mechanisms. The hyperinflammation and cytokine storm cause an excessive proliferation of T cells and monocytes, which can result in myocyte stress, injury and apoptosis, as well as subsequent fibroblast activation and extracellular matrix remodeling, ultimately resulting in diffuse myocardial and endothelial injury [41,42]. The underlying hyperinflammatory state is also a hypercoagulable state which can cause the development of coronary microvasculature thrombosis [20,41,43]. This high stress state can also cause any pre-existing coronary plaques to rupture and form additional thrombi. There is also evidence of direct viral invasion of both the myocardium and endothelium, which have been noted on biopsy and autopsy results [42,44]. Another mechanism of injury is systemic secondary damage to the myocardium and the cardiovascular system, which can be from respiratory failure and hypoxemia due to COVID pneumonia or ARDS or a diffuse hypercoagulable state resulting in decreased perfusion of the myocardium.

Through the various mechanisms of myocardial damage discussed, elevated cTn in COVID-19 patients has been noted to occur largely via three primary pathologies: myocarditis, microangiopathy and myocardial infarction (MI). Myocarditis can occur either by a direct viral invasion and damage to the cardiomyocytes resulting in a T-cell response and subsequent inflammation of the myocardium or by an indirect inflammation due to the cytokine storm response to the infection [40,45,46]. On biopsy, high viral load or diffuse inflammatory monocular infiltrates may be seen in the myocardium. Microangiopathy can similarly occur due to the direct viral invasion of the endothelium via ACE2 found on the endothelium resulting in vasculature injury. It can also occur indirectly via the hypercoagulable and prothrombotic state in these patients resulting in endothelial dysfunction and ultimately myocardial damage due to perfusion defects, vessel hyperpermeability and vasospasms [47]. Perhaps the currently most elucidated cardiovascular complication of COVID-19 is MI. Past studies of patients during the influenza seasons have similarly shown that patients with severe influenza symptoms had a significantly increased risk of MI due to the hyperinflammatory state as a result of the virus, which resulted in proliferation of proinflammatory cytokines, macrophage infiltration of the arterial walls, and subsequent prothrombotic state resulting in myocardial injury and ultimately MI [48,49]. Similar to the pathogenesis of myocarditis and microangiopathy, MI in COVID-19 patients can occur either due to direct or indirect damage to the myocardium [40,45]. Direct damage can occur via direct viral invasion and indirect hyperinflammatory state, like what has been seen in influenza patients, resulting in plaque instability and rupture with subsequent thrombosis. Indirect damage and subsequent MI are largely due to hypoxemia due to the underlying lung pathology; however, there is evidence that fever, tachycardia, or endocrine dysregulation, all of which is prominent in COVID-19 patients, can also result in hypoxemia. Several of the key studies cited here that may be important further consider are listed Table 1.

**Table 1.** List of key cited studies for further consideration.

Author	Title	Ref.
Driggin Elissa *et al.*	Cardiovascular considerations for patients, healthcare workers and health systems during the COVID-19 pandemic	[20]
Gaze DC	Clinical utility of cardiac troponin measurement in COVID-19 infection	[33]
Imazio M *et al.*	COVID-19 pandemic and troponin: indirect myocardial injury, myocardial inflammation or myocarditis?	[40]
Tersalvi G *et al.*	Elevated troponin in patients with Coronavirus Disease 2019: possible mechanisms	[45]
Kawakami R *et al.*	Pathological evidence for SARS-CoV-2 as a cause of myocarditis	[46]
Bikdeli B *et al.*	COVID-19 and thrombotic or thromboembolic disease: implications for prevention, antithrombotic therapy and follow-up	[47]
Guzik TJ *et al.*	COVID-19 and the cardiovascular system: implications for risk assessment, diagnosis and treatment options	[50]

COVID-19: Coronavirus disease 2019; SARS-CoV-2: Severe acute respiratory syndrome coronavirus 2.

Briefly mentioned above, there has been evidence of COVID-19-associated myocardial damage presenting similarly to what was previously seen in patients with influenza-associated myocardial disease. Due to the wide-range of clinical complications of COVID-19 contributing to the mortality rates, along with the many underlying comorbidities these patients may have, it is very difficult to accurately compare the mortality rates specifically due to CVD secondary to influenza and COVID-19. However, knowledge from past influenza seasons can serve as a key model in myocardial damage seen in COVID-19 patients by considering whether there are notable similarities to past viral pandemics or the annual influenza. Past studies of influenza have shown a significant correlation between seasonal influenza incidence and cardiovascular mortality, mainly MI and ischemic heart disease [48,51,52]. Study of the mechanism of myocardial damage in these patients showed transient endothelial dysfunction, which in turn destabilized vulnerable atherosclerotic plaques, increasing the risk of acute MI [48]. There was also evidence of an overactivation of inflammatory and coagulation pathways due to the overexpression of inflammatory cytokines, infiltration of arterial and myocardial walls by macrophages, and indirect damage to vascular endothelium due to a hypercoagulable state. A study of the pandemic (H1N1) 2009 virus infection similarly showed a strong association between acute myocardial dysfunction and elevated cTns [53]. Biopsies of the myocardium of patients with the H1N1 viral infection also showed microscopic foci of lymphoid aggregates with myofibril necrosis, comparable to the diffuse inflammatory monocular infiltrates that could be seen in the myocardial biopsies of COVID-19 patients. It is thus important to reassess whether the current findings of myocardial damage and subsequent complications are in fact significantly different from that of past pandemics. More importantly, however, this similarity provides insight into both how the current management of COVID-19 patients does not significantly vary from the treatment of critically ill patients in previous pandemics, and the overall success in being able to manage and treat patients with COVID-19 and CVD complications.

## Troponin as a prognostic factor

As the pathogenesis of cTn elevation due to myocardial injury in COVID-19 is better understood, it is important to consider the potential benefit of measuring cTn in COVID-19 patients. As discussed, pre-existing cardiovascular health is a powerful predictor of disease severity following SARS-CoV-2 infection and more specifically, the presence of myocardial injury is associated with an increased risk of complications and death in COVID-19 patients [50,54]. Therefore, it is necessary to consider the most effective methods of quantifying and representing the level of myocardial injury in patients. Pro-inflammatory markers such as N-terminal-pro brain natriuretic peptide, elevated cTn, and elevated high-sensitivity CRP are known markers of myocardial injury, and recent international guidelines for the diagnosis of myocardial damage and acute MI emphasize the value of measuring high-sensitivity cardiac troponin T (hs-cTnT) and cardiac troponin I (hs-cTnI) [55–57]. Within the context of COVID-19, while cTnT levels have shown promise as a prognostic factor for severity of illness, cTnI has been the more commonly reported metric for virus-related cardiac injury in COVID-19 patients [58,59].

Many studies have shown evidence of significantly elevated hs-cTnI in COVID-19 patients. A study of 700 COVID-19 patients at the University of Pennsylvania hospital reported elevated troponin concentrations for ICU patients upon admission [60]. In a separate study of 113 patients in Italy, patients were stratified into different groups depending on the serum level of hs-cTnI, and it was noted that patients in the group with the highest level of hs-cTnI had a longer hospital stay (median 37 days) and a greater need for ICU admission [61]. Additionally, in a study completed in Illinois, 673 patients with elevated troponin had significantly increased odds of critical illness (OR 3.65; 95% CI 2.03–6.57) [62]. Not only is cTnI an independent predictor of disease severity and ICU admission, but it is also, unsurprisingly, associated with increased patient mortality [63–65]. For example, in a study of 1919 patients in Wuhan, patients with acute cardiac injury, defined as serum hs-cTnI above the 99th percentile upper reference limit, had an odds ratio of 80.07 for in-hospital mortality, and nonsurvivors had significantly higher levels of hs-cTnI (nearly tenfold) than survivors [66]. The relevance of cTnI is further highlighted when considering that after adjusting for relevant clinical factors, even small amounts of myocardial injury were associated with a significantly increased risk of patient mortality, and that elevated levels of cTnI remain an independent predictor of death regardless of other elevated acute phase proteins and inflammatory markers in patients with CVD [67]. It is also important to note the prognostic value of cTnI in comparison to other inflammatory biomarkers such as CRP, D-dimer and lactate dehydrogenase. In a recent study comparing the short-term prognostic values of the above mentioned biomarkers, increased levels of all these inflammatory markers were associated with increased short-term mortality and increased risk of all-cause death in COVID-19 patients [68]. However, as a predictor for 30-day all-cause death, cTnI was a significantly better predictor compared with CRP, lactate dehydrogenase and D-dimer and additionally, cTnI as low as 21 ng/l was able to provide excellent prediction capacity.

In summary, cTnI is a biomarker highly useful for measuring myocardial injury and is considered a gold standard for the early diagnosis of cardiac complications such as MI, microangiopathy or myocarditis with great clinical relevance [55,69]. Furthermore, as pre-existing cardiovascular health is a good predictive factor for risk and severity following SARS-CoV-2 infection, and serum cTnI additionally is an independent predictor of COVID-19 disease severity and mortality, it is critical to consider the role of cTnI in COVID-19 risk stratification [64,70]. In studies of patients with cardiac injury, defined as cTnI levels above the 99th percentile, cardiac injury was significantly related to a lower survival rate, further emphasizing the relationship between myocardial health and COVID-19 severity [71,72]. Naturally, on patient admissions, one can measure a variety of biomarkers such as cTnT, N-terminal-pro brain natriuretic peptide, alanine transaminase, D-dimer or CRP, as determiners of inflammatory status; however, cTnI has been reported with the greatest prognostic value for patient risk and is thus the most promising agent for the hopeful identification of patients who would develop the most severe systemic inflammatory response to SARS-CoV-2 infection and subsequent cardiac complications, which can be fatal.

## Conclusion & future perspective

Not only is CVD a significant risk factor for worse clinical outcome in COVID-19 patients, but studies have also suggested the onset of new myocardial injury and cardiac pathologies due to the infection with SARS-CoV-2, as suggested by significantly elevated cTn. As discussed, there are many pathologic mechanisms of injury to the myocardium whether it is due to direct viral invasion into the myocardium and endothelium or damage to the myocardium due to the underlying hyperinflammatory, hypercoagulable and prothrombotic state or indirect damage due to systemic disease, subsequent hypoxemia and poor myocardial perfusion. While new onset MI has been most seen in COVID-19 patients, the risk of myocarditis and microangiopathy remains high and is of serious concern in patients who are already critically ill. As a prognostic factor for these patients, cTn, and specifically cTnI, have significant value in predicting the clinical outcomes in these patients and is an independent predictor of disease severity, ICU admission, and mortality in COVID-19 patients. This is especially important for patients with underlying inflammatory states such as chronic illnesses or end-stage renal disease, as baseline cTnI can already be elevated and these patients are at an increased risk of severe disease.

Thus, moving forward in the care of COVID-19 patients, it is imperative to monitor cTnI levels in hospitalized patients to identify and manage any clinical or subclinical myocardial inflammation prior to the development of major cardiac pathologies such as myocarditis, microangiopathy and MI, and to allow for a more specific and prompter therapy to be undertaken. As noted previously, due to a significant proportion of the population with underlying comorbidities experiencing an increased risk for severe COVID-19 illness, this current review of cTns in COVID-19 patients can provide insight on the vulnerability of select populations to severe illness. The number of vaccinated people has continued to increase nationally, but there is still hesitance among a significant portion of the country, thus challenging the goal of effectively lowering the number of COVID-19 infections both nationally and globally. It would be imperative for future studies to see how the vaccinations or lack thereof impacted these vulnerable populations so that we can better understand the efficacy of the vaccine and perhaps better understand the pathogenesis of COVID-19.

**Executive summary**

- Cardiovascular diseases (CVD), hypertension, Type 2 diabetes mellitus, cancer, chronic kidney diseases, congestive heart failure, obesity and smoking are associated with significantly increased severity of coronavirus disease 2019 (COVID-19) infection.
- Increased levels of cardiac troponin have been correlated with higher rates of CVD complications and mortality in COVID-19 patients.
- Risk of severe COVID-19 illness is increased for patients with pre-existing CVD.
- Primary pathological mechanism of injury to myocardium is through direct viral invasion into the myocardium and endothelium or damage to the myocardium due to the underlying hyperinflammatory, hypercoagulable and prothrombotic state.
- Secondary pathological mechanism of injury to myocardium is through indirect damage due to systemic disease, subsequent hypoxemia and poor myocardial perfusion.
- Cardiac troponins can be an effective biomarker for measuring myocardial injury in COVID-19 patients and can be an independent predictor of COVID-19 disease severity and mortality and has shown greater prognostic value for at risk patients compared with other inflammatory biomarkers.
